# Shisha Smoking Practices, Use Reasons, Attitudes, Health Effects and Intentions to Quit among Shisha Smokers in Malaysia

**DOI:** 10.3390/ijerph13070726

**Published:** 2016-07-19

**Authors:** Li Ping Wong, Haridah Alias, Nasrin Aghamohammadi, Sima Aghazadeh, Victor Chee Wai Hoe

**Affiliations:** 1 Julius Centre University of Malaya (JCUM), Department of Social and Preventive Medicine, Faculty of Medicine, University of Malaya, 50603 Kuala Lumpur, Malaysia; fi8erbreathing@gmail.com (H.A.); victorhoe@um.edu.my (V.C.W.H.); 2 Centre for Occupational and Environmental Health, Department of Social and Preventive Medicine, Faculty of Medicine, University of Malaya, 50603 Kuala Lumpur, Malaysia; nasrin@ummc.edu.my; 3 Innovative International College, Petaling Jaya, 46000 Selangor, Malaysia; aghazadehsima@gmail.com

**Keywords:** shisha, practices, reason for use, attitudes, health effects and intention to quit

## Abstract

Despite its popularity, shisha smoking practices, reasons for its use, attitudes, detrimental health effects and intention to quit among shisha users in Malaysia have never been investigated. A total of 503 shisha users responded to a cross-sectional study conducted between July 2015 and March 2016. The majority of users were young people aged 21–30; a small minority were underage. The reasons for shisha use were its growing popularity as a favourite pastime activity and the perception of shisha use as cool and trendy. Just over half (57.3%) agree that shisha use exposes the smoker to large amounts of smoke and the majority were unsure about the health risks of shisha smoking compared to tobacco smoking. The three most common detrimental health effects reported by the study respondents were dry throat, headache and nausea. Regular shisha users have significantly higher detrimental health effects compared to no-regular shisha users. Shisha users with a duration of smoking of 6–12 months (odds ratio (OR) 3.212; 95% confidence interval (CI) 1.651–6.248) and 6 months and below (OR 2.601; 95% CI 1.475–4.584) were significantly more likely to have a higher proportion who intend quitting smoking than shisha users of more than 12 months duration.

## 1. Introduction

Shisha is becoming an increasingly popular method of tobacco use worldwide. It originated in the Eastern Mediterranean region and is now gaining popularity in many western countries including Australia, the UK, Canada, and the USA, and also in Southeast Asia. Shisha is typically smoked in social settings such as cafés and restaurants, and is very frequently smoked by urban youth, young professionals, and university and college students [1,2,3]. Likewise, in Malaysia shisha smoking is gaining popularity nationwide. Many restaurants in urban areas are now offering shisha to their customers and it has become a new social scene. It has been reported that shisha smoking is even prevalent among medical students in Malaysia [4].

As a result of the rising popularity, shisha smoking is a growing threat to public health. The reason is because, firstly, there is a common misconception that smoking shisha is relatively less hazardous than smoking tobacco cigarettes, and secondly, most of outlets offering shisha remain largely unregulated. The erroneous perception that shisha is less hazardous than tobacco cigarettes has been a very widespread view [5,6,7,8,9,10]. Many erroneously believe that bubbling tobacco smoke through water makes it safe [11]. However, recent research proved that filtering tobacco smoke through water does not make it safer [12]. There is mounting evidence that indicates that shisha smoking is even more harmful than tobacco cigarettes [13,14]. Shisha smoking takes place typically in groups and lasts for nearly an hour. Therefore, shisha smokers often inhale more smoke than tobacco cigarette smokers because of the length of time a shisha session lasts. Furthermore, all members in the group will be exposed to the hazard of second-hand smoke from other members in the group in addition to their own shisha smoke. In addition to exposure to smoke from the tobacco, shisha smokers are also exposed to other toxic substances such as charcoal from the heat source [15]. Besides, as shisha is commonly served in cafes and restaurants, second-hand smoke exposure from shisha can be a health risk for non-smokers who are present in these venues.

In a similar way to tobacco cigarettes shisha has been shown to be associated with a wide range of detrimental health effects such as cancers, heart disease, lung disease and many other illnesses. A review of the health effects of smoking shisha synthesised that shisha smoking leads to significant exposure to polycyclic aromatic hydrocarbons (PAHs), volatile aldehydes, carbon monoxide, nitric oxide, nicotine, furans and nanoparticles. These agents have a wide range of harmful health effects ranging from cancer to respiratory diseases [16]. Additionally, the common practice of sharing a water-pipe mouth-piece poses a serious risk of transmission of communicable diseases including tuberculosis, hepatitis A and many others. The water inside the shisha apparatus may be contaminated, which can result into the spread and transmission of diseases [17,18].

Despite the rising popularity of shisha smoking among Malaysians, to-date, little research has been done on shisha use in Malaysia. There has been a study on shisha smoking among a small group of medical students [4]. However, a study on shisha smokers among the general public in Malaysia is lacking. The present study aimed to investigate the profile of shisha smokers generally seen at shisha lounges or restaurants that serve shisha. Specifically we sought to investigate their shisha smoking practices, their attitudes towards shisha smoking, the reasons for their shisha use and their self-reported detrimental health effects due to shisha smoking. The intention to quit smoking shisha and the associated factors will also be explored. Understanding who intends to stop by using e-cigarettes would allow for distinguishing user types and allowing future public health campaigns to focus on this subtype of shisha user.

## 2. Materials and Methods

### 2.1. Study Respondents and Settings

This cross-sectional study was conducted between July 2015 and March 2016. Researchers approached shisha users at shisha lounges or restaurants that serve shisha in the city of Kuala Lumpur and the area of Klang Valley in Selangor state, Malaysia. The researchers explained the purpose of the study and participations were voluntary. After respondents had read and understood the study information sheet, written informed consent was obtained and they were given the self-administered questionnaire.

### 2.2. Study Questionnaire

The researchers developed a semi-structured questionnaire based on a literature review and discussion among the research team members. The questionnaire was then pilot tested and revised before being administrated. The questionnaire consisted of 30 items divided into six sections. The first section assessed the respondents’ socio-demographic background. The second section included three items that assessed the respondents’ practices of using shisha. The third section included nine items to determine the reasons for using shisha. The fourth section included seven items that assess attitudes towards shisha smoking. The fifth section was about the experience of detrimental health effects associated with using shisha. The last section investigated the intention to quit shisha.

### 2.3. Data Analyses

Chi-squared and Fisher’s exact test were used to examine univariate association between categorical variables and outcome of interests, namely the total detrimental health effects and intention to quit smoking shisha. Multivariate logistic analysis was used to examine factors associated with the intention to quit shisha. In the multivariate logistic analysis, all variables with *p* < 0.05 in the univariate analysis were entered as a single block into the model (simultaneous forced entry). Adjusted odds ratio (OR) and 95% confidence interval (CI) were calculated. All analysis was performed with IBM SPSS Statistics version 19.0 (IBM, Armonk, NY, USA).

### 2.4. Ethical Consideration

The study was approved by the University Malaya Medical Ethics Committee (MECID NO: 20148-456). Informed consent for the interview was obtained from the respondents.

## 3. Results

In total, 503 completed responses were obtained and the response rate was 76.5%. Table 1 shows the socio-demographic characteristics and shisha smoking practices among the respondents. The oldest respondent was 64 years old and the youngest was 15 years old. Most of the respondents were 21–30 years of age (*n* = 317, 63.0%). There were 1.2% (*n* = 6) underage respondents (below 18 years old). The majority of the respondents were males (*n* = 403, 80.1%) and were single (*n* = 341, 67.8%). Most of the respondents were Malay (*n* = 377, 75.0%).

**Figure 1:**
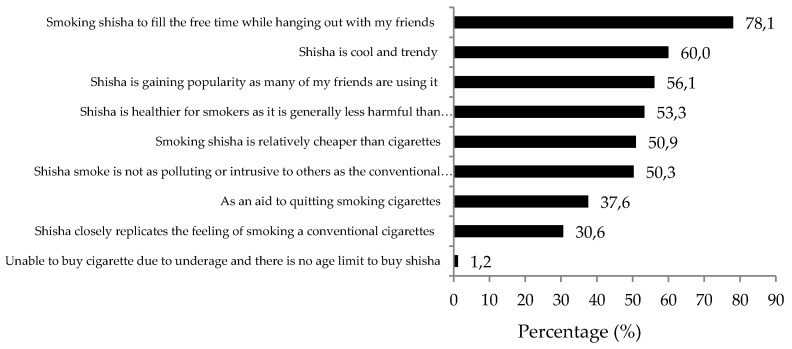
Reasons for smoking shisha (*N* = 503).

The majority had a tertiary education (*n* = 330, 65.6%). For the distribution of respondents by type of occupation, most of the respondents were skilled or non-skilled workers (*n* = 226, 44.9%), followed by students (*n* = 148, 29.4%). The majority of respondents reported having an average monthly income of RM1000 (one Malaysian Ringgit is equal to USD$0.25) and below (*n* = 169, 33.6%). Of the total study sample, only 18.1% (*n* = 91) were regular shisha smokers. The majority of the study respondents had been smoking shisha for 6 months or less (*n* = 236, 46.9%), and 33.6% (*n* = 169) had been smoking shisha for more than 12 months. Near two thirds (*n* = 302, 60.4%) smoke shisha once a week or less (54.8%, *n* = 318), and 40% smoke shisha twice a week. When the study respondents were asked about the reason they smoke shisha, the most common answer was to fill free time while hanging out with friends (78.1%, *n* = 393) (Figure 1). The second was that shisha is cool and trendy (60.0%, *n* = 302) and followed with shisha is gaining popularity and many of their friends are also smoking shisha (56.1%, *n* = 282). Slightly over half thought that shisha is healthier and less harmful than tobacco cigarettes (53.3%, *n* = 268). Half of the respondents thought that shisha smoke is not as polluting or intrusive as cigarette smoke (50.3%, *n* = 253). All the underage participants reported being unable to buy tobacco cigarettes due to being underage and smoking shisha because there is no age limit imposed by the outlet operators.

**Figure 2:**
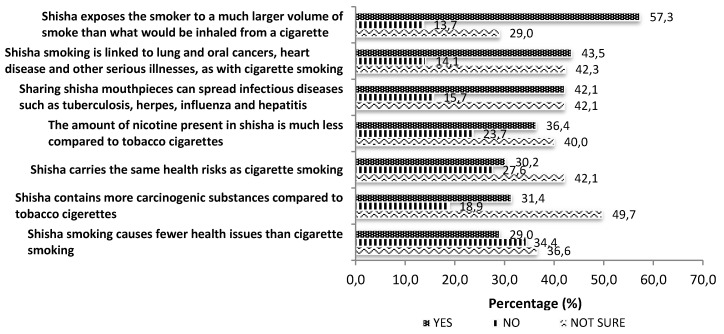
Attitudes towards shisha usage (*N* = 503).

In regards to attitudes to shisha smoking (Figure 2), over half (57.3%, *n* = 288) agreed that shisha exposes the smoker to a much larger volume of smoke than conventional tobacco cigarettes. Nearly half (42.3%, *n* = 213) were unsure if shisha is linked to diseases similar to those linked with smoking tobacco cigarettes, and if sharing shisha can spread infectious diseases (42.1%, *n* = 212). Slightly over one third (36.4%, *n* = 183) perceived that the amount of nicotine present is shisha smoke is less than in a tobacco cigarette, and a larger proportion 40% (*n* = 201) answered that they were unsure. The majority was unsure if shisha carries the same health risk as cigarette smoking (42.1%, *n* = 212), or if shisha contains more carcinogenic substances compared to tobacco cigarettes (49.7%, *n* = 250). A relatively higher proportion disagreed that shisha causes fewer health issues than cigarette smoking (34.4%, *n* = 173) compared to those that agreed (29.0%, *n* = 146).

**Figure 3:**
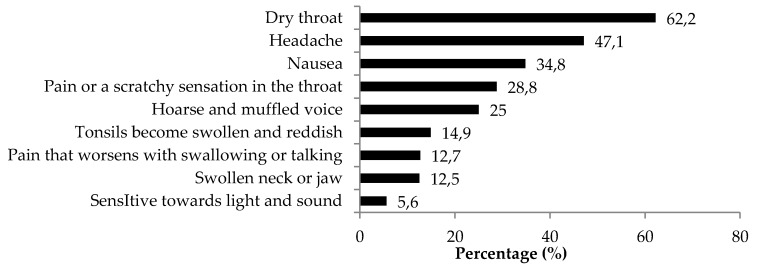
Adverse health effects associated with shisha use (*N* = 503).

As shown in Figure 3, the three most common detrimental health effects reported by the study respondents were dry throat (62.2%, *n* = 313), followed by headache (47.1%, *n* = 237), and nausea (34.8%, *n* = 175). It is noteworthy that, among the six underage respondents, four respondents reported experiencing dry throat, and two respondents reported experiencing headache and nausea, respectively. All six underage shisha users reported experiencing at least one detrimental health effect. The median (interquartile range) of number of detrimental health effects was 2 (IQR 1 to 3). Based on the median value as a cut-off point, the total number of detrimental health effects was divided into two groups and associated with shisha smoking practices (Table 2). Shisha smoking status was the only significant factor associated with the total number of detrimental health effects. Regular smokers had higher odds of having number of detrimental health effects of 3–9 versus 0–2 (OR = 2.264, 95% CI: 1.42–3.60, *p* < 0.01) than non-regular smokers. Although not statistically significant, respondents with a duration of smoking above 12 months reported the highest proportion of total detrimental health effects 3–9 (46.2%) compared to those who smoked for 6–12 months (41.8%) and 6 months or less (43.2%). Although statistically not significant, respondents with a duration of smoking above 12 months recorded a higher proportion of total detrimental health effects of 3–9.

In total, 290 respondents (57.6%) expressed the intention to quit smoking shisha. Table 3 shows the proportion with the intention to quit smoking shisha by demographic characteristics, shisha smoking practises, reasons for shisha smoking and total detrimental health effects. In the multivariate analysis, the duration of smoking was significantly associated with the intention to quit smoking shisha. Respondents with a duration of smoking shisha of 6–12 months (OR 3.212; 95% CI 1.651–6.248) and 6 months and below (OR 2.601; 95% CI 1.475–4.584) were significantly more likely to have a higher proportion of respondents with the intention to quit smoking shisha than the reference group of those with a smoking duration above 12 months.

Some of the reasons for shisha smoking were found to be significantly associated with the intention to quit smoking shisha in the multivariate model. Respondents who use shisha because of the perception that shisha is healthier than tobacco cigarettes were less likely to quit shisha (OR 0.384; 95% CI 0.232–0.635). Respondents who use shisha because they are unable to buy tobacco cigarettes due to being underage (OR 2.217; 95% CI 1.340–3.669) have a significantly higher level of intention to quit smoking shisha. Respondents who use shisha because shisha is not as polluting as tobacco cigarettes (OR 0.521; 95% CI 0.317–0.858), who think that shisha is cool and trendy (OR 0.405; 95% CI 0.244–0.672), and that shisha is relatively cheaper than tobacco cigarettes (OR 0.483; 95%CI 0.297–0.784), were less likely to quit shisha smoking.

## 4. Discussion

A large proportion of our study respondents (76.1%) were aged 30 and younger. This may imply that the majority of shisha users were young adults as has also been found to be the case in many other studies [10,19,20]. Although the proportion of women using shisha is relatively lower than the proportion of men, there is a growing concern about shisha use among women in Malaysia, especially because the majority of them are of reproductive age. Tobacco use causes many unique risks to women, namely higher rate of infertility, premature birth, infants born at low birth weight, ectopic pregnancy, infant mortality, cervical cancer, irregular menstruation, dysmenorrhea and premature menopause [21,22]. The growing popularity of shisha use suggests that, in Malaysia, the existing anti-smoking campaigns and advisements, which target mainly tobacco cigarettes, should also include shisha smoking as well as other nicotine delivery systems that are gaining popularity such as e-cigarettes. Furthermore, the popularity of females using shisha implies that the health risks awareness health message that often focuses on lung cancer as a health effect of smoking should now also emphasise the health effects of smoking on women.

In line with studies conducted elsewhere [23,24], this study provides evidence of underage shisha smoking. Although only six respondents were of age 15–18 years, this is a worrying concern as it may indicate that shisha outlets do not prevent sales to those who are underage. It is time to bring the problem of teen shisha smoking to the attention of the nation. Shisha retailers should play an essential role in prohibiting teen access to shisha smoking. In Malaysia, as yet, there is no legislation to prevent those who are underage going to shisha serving outlets and accessing shisha. It is important that legislation to prohibit underage access to tobacco should also include restrictions for shisha outlets selling to those who are underage.

It is of concern that nearly 30% of the study respondents were students. Apart from six underage secondary school users, the remaining were college and university students. Worldwide, many studies on shisha smoking reported that shisha smoking is popular among college and university students [25,26,27,28]. A study found that the prevalence of trying out shisha and shisha smoking increased with the duration in the university, which might indicate that shisha smoking is propagated through student culture [25]. These findings suggest a need for an increased emphasis on anti-shisha smoking programmes in college and university to discourage shisha smoking. Awareness messages should highlight the negative long term health consequences of adolescent or youth smoking because young people may feel that they are invulnerable to smoking related health risks [29].

The reasons for smoking shisha identified in this study provide important information as well as an explanation for the current situation of shisha smoking in Malaysia. Firstly, the top three most common reasons for smoking shisha are: to fill free time while hanging out with friends, shisha is trendy and cool, and shisha is gaining popularity among their peers, implying that shisha smoking appears to be the result of social norms or common social behaviours. It appears that shisha smoking has become a situational norm and has influenced and encouraged many belonging to the same social group to adopt the behaviour. This finding implies that the social norms approach should be used in Malaysia to address the rising shisha smoking trend. Social norms marketing interventions have been successfully used to reduce or to promote quitting smoking and alcohol use among college students [30,31,32]. In this study, all underage participants reported that one of the reasons they smoke is because they are unable to buy tobacco cigarettes and they are not restricted from access to shisha. The evidence is clear that those who are underage resort to shisha as there is a restriction on buying tobacco cigarettes. Therefore, there is an urgent need for strict enforcement of regulations for outlets offering shisha to prohibit selling shisha products to underage persons. Shisha should be subjected to the same regulation as cigarettes and other tobacco products to limit access of underage users. Failure to impose strict control may encourage those who are underage to turn to shisha as an alternative to tobacco.

Understanding respondents’ attitudes towards shisha smoking provides important implications for the future direction of intervention to reduce shisha smoking. Just over half of the respondents believed that the shisha smoker is exposed to a massive volume of smoke. This shows the need to enlighten shisha smokers regarding the fact that the shisha user may be exposed to a much higher volume of smoke and thus to higher exposure to cancer causing chemicals and hazardous gases such as carbon monoxide. It was reported that a shisha smoker may inhale up to 200 times more smoke in a single session as compared to a cigarette smoker [33]. The most common response given by our study respondents when asked about the health risk perception of shisha smoking was “unsure” implying a lack of knowledge of the health risks associated with shisha smoking. Many were also unsure when asked if shisha smoking is healthier and causes relatively fewer health issues than tobacco cigarettes. Near 30% of our study respondents viewed shisha as healthier, implying that our study participants share the same opinion as reported in other studies where the common misperception is that the shisha smoke is filtered by water and is less hazardous. Evidence that even after passing through water, tobacco smoke still contains high levels of carcinogens, including carbon monoxide [20], should be made known to shisha users. Therefore shisha smokers should be made aware that shisha is not less harmful than tobacco cigarettes.

The three most common detrimental health effects reported by the study respondents, namely dry throat, headache and nausea were in accordance with many established reports [34,35]. It is a matter of concern that over half (62%) of our study respondents reported experiencing dry throat as a result of using shisha. The study also reveals worrying facts about the health consequences of underage shisha smoking. The underage shisha users in this study reported health effects due to using shisha similar to those reported by adult shisha users. It is of concern that, despite their young age, all experienced at least one adverse health effect. It is timely that adolescents should be exposed to anti shisha smoking messages from schools, families and mass media. They should be informed that cigarette smoking during childhood and adolescence causes a range of immediate health problems, as well as laying the foundation for the development of serious diseases in adulthood [36]. Furthermore, due to the adverse effects of nicotine on brain development, chronic exposure to nicotine may have harmful effects, particularly on foetal development and among young people. In addition, smoking cigarettes during adolescence has been associated with lasting cognitive and behavioural impairments, including effects on working memory and attention, and reduced prefrontal cortex activation [22]. As such, shisha prevention messages should include the fact that not only are adolescent shisha users exposed to similar health risks as adult users, but they are even more vulnerable to the adverse effects of smoking than adults are.

It is of note that the majority of the overall study participants reported having at least two detrimental health effects reflecting the immense impact of shisha on users’ health. In particular, regular shisha users have significantly (two-fold) higher odds of high total detrimental health effects. This may imply that the frequent use of shisha increases the amount of exposure to the hazards of shisha smoke. More frequent shisha smoking may imply increased levels of inhaled nicotine and carcinogens absorbed by the body that lead to detrimental health effects. Therefore, shisha users should be given the message that smoking shisha regularly is the most important predictor of a high number of detrimental health effects.

In this study, only slightly over half of the respondents have the intention to quit shisha smoking. This may imply that more efforts need to be made to increase the chances of a shisha smoker stopping smoking. Our multivariate analysis identified important factors that are significantly associated with the intention to quit using shisha and may provide an insight into efforts at shisha control and intervention to encourage quitting shisha use. A particularly noteworthy finding from the multivariate analysis was the longer a person smokes shisha, the less likely a person is to want to quit. In general, nicotine addiction is associated with the length of time one has been a smoker. Increased nicotine dependence was reported to be associated with difficulty in quitting tobacco smoking [37]. Hence, our results indicate that efforts aimed to helping shisha users to quit should start when a person has just started smoking shisha. Respondents who use shisha because of the perception that shisha is healthier than tobacco cigarettes and that shisha smoke is not as intrusive as tobacco cigarette smoke were less likely to quit shisha. This implies that a programme raising awareness and educating shisha users should specifically include messages that shisha use can be as harmful, or even more harmful, than the use of tobacco cigarettes, and that shisha smoke is not non-intrusive to other people or in public places.

Underage respondents, who were unable to buy tobacco cigarettes due to their age, have a significantly higher number with the intention to quit smoking shisha. This implies that underage respondents have intention to quit and that adequate support should be provided for them to increase their likelihood of successfully quitting shisha. School-based smoking prevention programmes are reported to be one of the most effective strategies to reduce smoking among adolescents, and have been shown to reduce smoking intention and behaviour [38,39,40,41]. These findings suggest that future health promotion activities in schools in Malaysia should include a comprehensive tobacco awareness element that should include shisha smoking and other nicotine delivery devices such as electronic cigarette.

The non-significant association between total detrimental health effects and the intention to quit smoking shisha is of particular concern. It may indicate that detrimental health effects are of least concern when it comes to the decision to quit shisha smoking. This finding again emphasises the importance of the awareness of the health hazards of shisha smoking.

Due to the difficulty in getting a complete sampling frame of shisha users in Malaysia, we surveyed a convenience sample of shisha users at shisha lounges or restaurants that serve shisha in Kuala Lumpur and Klang Valley. The major limitation of this study is the reliance upon a convenience sample and upon self-reports. Therefore the results cannot be generalised to the entire population of shisha users in Malaysia.

## 5. Conclusions

In this study, shisha smoking was prevalent among young people and a small number of underage users was evident. The inquiry reveals that the reasons for shisha smoking appear to be the result of social norms or common social behaviours. Gaining insight into the respondents’ attitudes towards shisha smoking revealed the erroneous perception that shisha is healthier than tobacco cigarettes and that ignorance of the health hazards of shisha was common. In spite of the fact that most respondents experienced detrimental health effects due to shisha use, health effects are not of concern when it comes to the intention to quit shisha. In contrast, significant factors associated to the likelihood of a person not intending to quit shisha were long duration of shisha use, the perception that shisha is healthier than tobacco cigarettes, and the perception shisha smoke is not as intrusive compared to tobacco cigarette smoke. Interventions to reduce the use of shisha should focus on eradicating the misconception that shisha is healthier than tobacco cigarettes and providing factual information about the health hazards of shisha use. There is a need for strict enforcement of the rules that requires retailers not to offer shisha to underage users. Shisha should contain health warnings similar to tobacco cigarettes.

## Figures and Tables

**Table 1:** Distribution of socio-demographic characteristics and shisha smoking practices (*N* = 503).

Details	*n* (%)
**(A) Socio-demographic data**	
**Age group**	
20 and below	66 (13.1)
21–30 years old	317 (63.0)
>30 years old	120 (23.9)
**Gender**	
Male	403 (80.1)
Female	100 (19.9)
**Marital status**	
Single	341 (67.8)
Married or previously married	162 (32.2)
**Ethnic**	
Malay	377 (75.0)
Chinese	67 (13.3)
Indian	32 (6.4)
Others	27 (5.4)
**Highest education attainment**	
Secondary and below	173 (34.4)
Tertiary (university level)	330 (65.6)
**Occupation**	
Professional & Managerial	79 (15.7)
Skilled/Non-skilled worker	226 (44.9)
Student	148 (29.4)
Retiree	8 (1.6)
Unemployed	42 (8.3)
**Monthly income**	
≤RM1000	169 (33.6)
RM1000–2000	120 (23.9)
RM2001–3000	124 (24.7)
>RM3000	90 (17.9)
**(B) Shisha smoking practices**	
**Shisha Smoking status**	
Non-regular smoker	412 (81.9)
Regular smoker	91 (18.1)
**Duration of smoking**	
6 months and below	236 (46.9)
>6 months to 12 months	98 (19.5)
>12 months	169 (33.6)
**Frequency of shisha smoking in a week**	
Once in a week or less	302 (60.4)
2–3 times a week	201 (40.0)

**Table 2:** Association between shisha smoking practices and detrimental adverse health effects (*N* = 503).

Details	*n* (%)	Total Detrimental Health Effects			Binary Logistic Regression for Total Adverse Health Effects 3–9 vs. 0–2
**Shisha smoking practices**		0–2	3–9	*p*-Value	OR (95% CI)
		(*n* = 282)	(*n* = 221)		
**Shisha Smoking status**					
Non-regular smoker	412 (81.9)	246 (59.7)	166 (40.3)	0.001	Reference
Regular smoker	91 (18.1)	36 (39.6)	55 (60.5)		2.264 (1.424–3.601) **
**Duration of smoking**					
6 months and below	236 (46.9)	134 (56.8)	102 (43.2)		
>6 months to 12 months	98 (19.5)	57 (58.2)	41 (41.8)	0.755	
>12 months	169 (33.6)	91 (53.8)	78 (46.2)		
**Frequency of shisha smoking in a week**					
Once a week or less	302 (60.4)	168 (55.3)	134 (44.4)	0.855	
2–3 times	201 (40.0)	114 (56.7)	87 (43.3)		

** *p*-value < 0.01.

**Table 3:** Association between socio-demographic characteristics, shisha smoking practices, reasons for using shisha, total adverse health effects and intention to quit smoking shisha (*N* = 503).

Details	n (%)	Intention to Quit Smoking Shisha			Multiple Logistic Regression for Yes vs. No
**(A) Socio-demographic**		Yes (*n* = 290)	No (*n* = 213)	*p*-Value	Adjusted OR (95% CI)
**Age group**					
20 and below	66 (13.1)	46 (69.7)	20 (30.3)	0.058	
21–30 years old	317 (63.0)	172 (54.3)	145 (45.7)		
>30 years old	120 (23.9)	72 (60.0)	48 (40.0)		
**Gender**					
Male	403 (80.1)	249 (61.8)	154 (38.2)	<0.001	1.483 (0.822–2.673)
Female	100 (19.9)	41 (41.0)	59 (59.0)		Reference
**Marital status**					
Single	341 (67.8)	202 (59.2)	139 (40.8)	0.334	
Married or previously married	162 (32.2)	88 (54.3)	74 (45.7)		
**Ethnic**					
Malay	377 (75.0)	242 (64.2)	135 (35.8)	<0.001	2.242 (0.828–6.069)
Chinese	67 (13.3)	21 (31.3)	46 (68.7)		0.653 (0.216–1.977)
Indian	32 (6.4)	14 (43.8)	18 (56.2)		1.323 (0.366–4.782)
Others	27 (5.4)	13 (48.1)	14 (51.9)		Reference
**Highest educational attainment**					
Secondary and below	173 (34.4)	113 (65.3)	60 (34.7)	0.013	0.988 (0.549–1.815)
Tertiary (University level)	330 (65.6)	177 (53.6)	153 (46.4)		Reference
**Occupation**					
Professional & Managerial	79 (15.7)	44 (55.7)	35 (44.3)	<0.001	
Skilled/Non-skilled worker	226 (44.9)	154 (68.1)	72 (31.9)		0.937 (0.343–2.560)
Student	148 (29.4)	68 (45.9)	80 (54.1)		1.240 (0.506–3.038)
Retiree	8 (1.6)	4 (50.0)	4 (50.0)		1.191 (0.419–3.386)
Unemployed	42 (8.3)	20 (47.6)	22 (52.4)		1.036 (0.174–6.177)
**Monthly income**					
≤RM1000	169 (33.6)	78 (46.2)	91 (53.8)	0.010	0.509 (0.211–1.224)
RM1000–2000	120 (23.9)	79 (65.8)	41 (34.2)		0.812 (0.361–1.825)
RM2001–3000	124 (24.7)	83 (66.9)	41 (33.1)		1.095 (0.534–2.246)
>RM3000	90 (17.9)	50 (55.6)	40 (44.4)		Reference
**(B) Shisha smoking practices**					
**Shisha Smoking status**					
Non-regular smoker	412 (81.9)	252 (61.2)	160 (38.8)	0.001	0.963 (0.506–1.832)
Regular smoker	91 (18.1)	38 (41.8)	53 (58.2)		Reference
**Duration of smoking**					
6 months and below	236 (46.9)	167 (70.8)	69 (29.2)	<0.001	2.601 (1.475–4.584) **
>6 months to 12 months	98 (19.5)	59 (60.2)	39 (39.8)		3.212 (1.651–6.248) **
>12 months	169 (33.6)	64 (37.9)	105 (62.1)		Reference
**Frequency of shisha smoking in a week**					
Once	302 (60.4)	200 (66.2)	102 (33.8)	<0.001	1.569 (0.929–2.650)
2 times and above	201 (40.0)	90 (44.8)	111 (55.2)		Reference
**(C) Reasons for using shisha**					
**Shisha is healthier for smokers as it is generally less harmful than conventional tobacco cigarettes**					
Yes	268 (53.3)	107 (39.9)	161 (60.1)	<0.001	0.384 (0.232–0.635) ***
No	235 (46.7)	183 (77.9)	52 (22.1)		Reference
**Shisha smoke is not as polluting or intrusive to others as the conventional tobacco cigarette**					
Yes	253 (50.3)	113 (44.7)	140 (55.3)	<0.001	0.521 (0.317–0.858) *
No	250 (49.7)	177 (70.8)	73 (29.2)		Reference
**Shisha closely replicates the feeling of smoking a conventional tobacco cigarette**					
Yes	154 (30.6)	74 (48.1)	80 (51.9)	0.004	0.840 (0.498–1.416)
No	349 (69.4)	216 (61.9)	133 (38.1)		Reference
**Unable to buy tobacco cigarettes due to being under age and there are no age limits on buying shisha**					
Yes	155 (30.8)	104 (67.1)	51 (32.9)	0.005	2.217 (1.340–3.669) **
No	382 (69.2)	186 (53.4)	162 (46.6)		Reference
**Shisha is cool and trendy**					
Yes	302 (60.0)	149 (49.3)	153 (50.7)	<0.001	0.405 (0.244–0.672) ***
No	201 (40.0)	141 (70.1)	60 (32.7)		Reference
**Shisha is gaining popularity and many of my friends are using it**					
Yes	282 (56.1)	130 (46.1)	152 (53.9)	<0.001	0.653 (0.395–1.078)
No	221 (43.9)	160 (72.4)	61 (27.6)		Reference
**Smoking shisha is relatively cheaper than smoking tobacco cigarettes**					
Yes	256 (50.9)	123 (48.0)	133 (52.0)	<0.001	0.483 (0.297–0.784) **
No	247 (49.1)	167 (67.6)	80 (32.4)		Reference
**Smoke shisha to fill free time while hanging out with my friends.**					
Yes	393 (78.1)	225 (57.3)	168 (42.7)	0.745	
No	110 (21.9)	65 (59.1)	45 (40.9)		
**As an aid to quitting smoking tobacco cigarettes**					
Yes	189 (37.6)	99 (52.4)	90 (47.6)	0.077	
No	314 (62.4)	191 (60.8)	123 (39.2)		
**(D) Symptoms experienced by shisha smokers**					
**0–2**	282 (56.1)	170 (60.3)	112 (39.7)	0.203	
**3–9**	221 (43.9)	120 (54.3)	101 (45.7)		

* *p*-value < 0.05; ** *p*-value < 0.01; *** *p*-value < 0.001.
